# Rethinking 2S/LGBTQI+ food security with co-design: a study protocol

**DOI:** 10.3389/fpubh.2025.1558700

**Published:** 2025-06-13

**Authors:** Joanie Thériault, Phillip Joy, Sana Boudhraâ, Min Gao

**Affiliations:** ^1^Department of Occupational Therapy, VITAM Centre de recherche en santé durable - CIUSSS Capitale-Nationale, Université du Québec à Trois-Rivières, Trois-Rivières, QC, Canada; ^2^Applied Human Nutrition, Mount Saint-Vincent University, Halifax, NSW, Canada; ^3^Expert in Codesign & Qualitative UX Research, The Laboratory of Research in Design: Hybridlab, Université de Montréal, Montréal, QC, Canada; ^4^Community Engagement Specialist, Innovation and Learning Lab, Feed Nova Scotia, Darthmouth, NSW, Canada

**Keywords:** food security, co-design, 2S/LGBTQI+, community-based research, participatory approach

## Abstract

**Context:**

In Canada, recent statistics show that 8.7 million Canadians face food insecurity which disproportionately affects people of the 2S/LGBTQI+ communities. Food insecurity is intersectional: people belonging to one or more marginalized groups, like 2S/LGBTQI+, are at greater risk. Moreover, food security resources can pose due to the stigma and cis-heterosexism associated with the religious basis of some of these resources. Exploring ways to partner up with and for 2S/LGBTQI+ communities and food security organizations in order to reflect and imagine a new service model is a promising avenue to tackle the social injustice of food insecurity.

**Objectives:**

This research protocol presents the activities and strategies of a co-design study aiming to enhance safety and inclusivity of food security services with and for 2S/LGBTQI+ individuals. The team also seeks to identify how to improve food security services with and for 2S/LGBTQI+ communities and to co-create a prototype service model representing safe and inclusive services that communities and food security stakeholders can utilize to make improvements in that direction.

**Methods:**

This protocol is based on a co-design methodology inspired by design thinking. The project will address desirability, feasibility, and viability - what is desirable, acceptable, achievable and sustainable in a prototype service model for 2S/LGBTQI+ individuals accessing food security services, organizations, and workers/volunteers. Participants will take part in seven online co-design workshops. Facilitators will guide the participants in offering free commentary, generating thoughts, and sharing new ideas along with reflective questions regarding a provisional prototype of the service model and framework principles. Discussions will be recorded for analysis purposes along with visual and textual content generated through the web-based collaborative tool. The data will be subjected to a qualitative thematic analysis.

**Conclusion:**

This protocol recognizes and values the experience and knowledge of 2S/LGBTQI+ communities and illustrates participatory involvement to improve food security. It is expected that this protocol inspires researchers and organizations to partner up and explore ways to use, replicate, and improve or adapt the approach. Future results may find interest and usefulness in other 2S/LGBTQI+ communities and food security organizations.

## 1 Introduction

Food insecurity is a contemporary social inequity that has now become a concern at the forefront of economic and social development. A specialized group in food security and policy research has positioned food insecurity “as the inadequate or insecure access to food due to financial constraints that is a serious public health issue, a marker of pervasive material deprivation, and a matter of public policy” (1). For 2023 in Canada, it is estimated that 8.7 million Canadians in the ten provinces lived in food insecurity (2). Researchers stress that these estimates are the highest in the almost 20 years of monitoring (1). Among the ten provinces, Nova Scotia has the highest percentage of individuals experiencing food insecurity with 28.9% (1, 2). Food insecurity particularly affects people from gender-diverse communities, people embodying different gender and sexual orientations that fall outside the dominant binaries of gender and sexual orientation (3–5), sometimes referred to as two-spirit, lesbian, gay, bisexual, transgender, queer, and others with the acronym 2S/LGBTQI+ (6). In Canada, individuals from 2S/LGBTQI+ communities experience a higher prevalence of food insecurity, with bisexual Canadians reporting rates three times greater than their heterosexual counterparts, regardless of their employment status (2, 7).

Food insecurity among 2S/LGBTQI+ individuals is associated with various economic barriers (8–10). Studies have demonstrated that 2S/LGBTQI+ individuals are more likely to have lower income and face employment issues, raising the need for food assistance (11, 12). These economic issues also have consequences on housing, limiting where people can live and, more importantly, where they feel safe enough to live (7, 11). Experiencing housing difficulties reduces the possibilities for 2S/LGBTQI+ individuals to live in areas where grocery stores are present, sometimes obliging them to live in food desert areas (13, 14). It has been recognized that at the roots of all these income, employment, and housing issues is the discrimination experienced by 2S/LGBTQI+ individuals (7–9, 13). In addition to discrimination, food insecurity adversely affects the mental and physical health of 2S/LGBTQI+ individuals. Many studies report that food insecurity is associated with infections, pain, chronic illnesses, and even premature mortality (7, 11, 14, 15). Research has shown that individuals from 2S/LGBTQI+ communities facing food insecurity exhibit elevated levels of depressive and anxiety symptoms, as well as higher incidences of eating disorders compared to cis-heterosexual individuals (16–18).

As a social response to food insecurity, various forms of services are offered to the population. One of the most renowned and prevalent forms is that of food banks, which were initially established as an emergency food service assistance or last resort solution (19, 20). Initially, many food banks were managed by charitable and/or faith-based organizations (21). Presently, food bank services are predominantly managed by non-profit civil organizations, yet charitable or faith-based organizations continue to play a role in food security, whether by offering space or a physical facility to operate the services (4, 20, 47). Emergency food service assistance, such as food banks, provided by the charitable food sector is regarded as being necessary, but it only serves a small percentage of citizens who are food insecure and lack the resources to meet continuous demands.

Many food bank organizations count on donations and volunteer workers to operate their services, which results in limited service hours (11). This constitutes important limitations that create barriers, especially for 2S/LGBTQI+ people. Some research highlights that 2S/LGBTQI+ individuals attending food banks re-experience stigma and micro-aggression like having to wait in line outside, receiving non-affirming services (4, 22, 23). The stigma and discrimination are particularly present in contexts of faith-based services, and this is particularly the case for individuals in nonurban regions (3, 4, 23). Additional barriers to safe and inclusive access to food banks for the general public, as well as for 2S/LGBTQI+ people, include the food’s quality and the inability to select items based on dietary restrictions, allergies, or intolerances (9, 11, 47). Despite some precited limitations, the food security area has deployed efforts to innovate in the different forms of services offered. Some food banks now offer a choice model service (people can select food from displays); some others offer additional services like recipe handouts, nutrition counseling, food literacy classes, and collective cooking groups (24).

Recently, other forms of food security services stemmed directly from communities. Community kitchens and group cooking where people can gather may help to improve cooking skills and foster networks and a sense of connectedness (11). While engaging in community kitchen activities may have socio-relational benefits (46), there is little evidence that these activities can reduce food insecurity, and there is a lack of information regarding specific programs for and with members of the 2/SLGBTQI+ community (48, 49). Local or community pantries and fridges where people can bring and take food in a mutual collaboration are other forms of food security initiatives. Community pantries and fridges may offer a more inclusive and dignified way to access healthy foods for 2S/LGBTQI+ populations, although very few seem to be specifically designed as such in Canada (4). Research underscores the potential for collaboration between 2S/LGBTQI+ community organizations and community food pantries and fridges to create a non-threatening and inclusive environment that more accurately addresses the needs of this population (10). Solidarity markets, or solidarity grocery stores, are innovative non-profit forms of services operating on a “pay what you can” basis, emphasizing promoting local foods and embodying solidarity, agency, and democracy (25). Again, solidarity markets offer a promising opportunity to represent the values and needs of humanity, respect, and inclusivity that are central to 2S/LGBTQI+ communities. More research is needed to determine how these service models are adapted for 2S/LGBTQI+ people (4).

Given this information, the potential for collaboration in social innovation between communities, food security organizations, and research remains insufficiently explored. Such collaboration would be even more valuable if participatory, namely that it should be undertaken with individuals from queer communities and other key stakeholders in the food security sector (5). Collaborating with individuals from 2S/LGBTQI+ communities to actively engage in rethinking food security services would allow for their experiential knowledge to be recognized, valued, and mobilized. The inclusion of people with experiential knowledge in collaborative work has benefits for the development of new health initiatives (26, 27). These reflect a better understanding of people’s experiences and needs and thus have the potential to be more useful and usable by the population (26, 27). Regarding food security service innovation, such an initiative would present an even more promising uptake if based on a research approach inspired by innovative and co-creative design, uniting all involved partners dedicated to enhancing food security services for, but mostly with 2S/LGBTQI+ individuals (5).

Aligning with these conclusions, this research protocol expands upon a previous community-based participatory research study that examined barriers to food accessibility with 2S/LGBTQI+ groups and their recommendations for culturally competent, safe, and inclusive services (5). Therefore, the objective of this research protocol is to present the research activities and strategies of a co-design study aiming to enhance safety and inclusivity of food security services with and for 2S/LGBTQI+ individuals. By conducting this study, the team seeks to concretely identify how to improve food security services with and for 2S/LGBTQI+ communities and to co-create a prototype service model representing safe and inclusive services that communities and food security stakeholders can utilize to make improvements in that direction.

## 2 Methods and analysis

### 2.1 Research approach

The research project adopts a co-design approach, a form of participatory co-creation activities and processes (28, 29). Mostly known for digital health solutions, co-design also applies to social innovations outside the digital domain. Experts have now reported co-design’s relevance in driving substantial contributions to social change and innovation (30, 31). Among the reasons for that is the collaboration with end users, who are not just acknowledged but actively co-create solutions together with designers, stakeholders, and researchers (29, 32). Potential end users are essential to any co-design approach, and the quality of their participation is of high importance since they are the experts in their own lived experience. End users actively participating in the co-design process of a social innovation enhance its capacity to meet their needs and may increase their engagement (33, 34). Additionally, co-design as a social interaction is based on a shared perspective of lived experience, rather than just gathering individual users’ experience. Battarbee and Koskinen (35), called this collective shared experience ‘co-experience’ and claimed that neglecting co-experience leads to a limited understanding of user experience and a similarly limited understanding of design possibilities. In this co-design research protocol, the end users involved are individuals from 2S/LGBTQI+ communities and individuals experiencing (or having experienced) food insecurity (or both), along with partners from the food security network, health services, and policy and regulation. A customizable model and framework principles for 2S/LGBTQI+ inclusive food security services present potential in social innovation.

The co-design approach of this project is inspired by design thinking approach (36). Design thinking is defined as a process used to resolve wicked problems or complex issues that are embedded in complex contexts that call for innovative solutions (37), and consists of a series of flexible and iterative steps, including the use of multiple methods and tools generating different artifacts (38). Design thinking involves play, empathy, reflection, creativity, and experimentation to facilitate collaboration, innovation, and the enhancement of discoveries (39). It enables individuals from different backgrounds to come together to address many societal issues and resolve them through a synthesis of diverse thinking methods that are human-centered (39). It allows generating innovative solutions based on a user-centered approach involving multidisciplinary teams (38). Three dimensions are considered in design thinking: desirability, feasibility, and viability. Design thinking is found relevant for social innovations centered on humans by combining what is humanly desirable with what is organizationally feasible and economically viable (36, 40). In the co-design approach of this study protocol, design thinking will be used with a major focus on desirability. Desirability will consider what is desirable and acceptable in this customizable model and framework principles with and for people of the 2S/LGBTQI+ communities accessing food security services and stakeholders involved in this area. Feasibility and viability will also be explored in consideration to how the desirable characteristics can be implemented in various organizations and how these can be sustained from a long term perspective.

### 2.2 Research team

The research team is composed of two principal researchers. They will work in partnership with members of a food security partner organization located in Nova Scotia. Members of the partner organization are a community planner and a community engagement specialist who was trained as a dietitian. The research team has experience on the topic of gender and sexuality within nutrition, dietetics, and health, along with experience in community-based participatory research framed within post-structural and social constructivism frameworks mobilizing individuals with lived experienced. All team members strive to contribute more knowledge to the experiences of sexual and gender-diverse individuals who experience food insecurity, with the goal of advocating for safer and more inclusive services. The team also includes members of the 2S/LGBTQI+ community.

### 2.3 Recruitment and participants

After obtaining ethics clearance, a call for participation will be launched through the team’s channels (social media, mailing list etc.) to form co-design groups with people presenting mixed and diverse expertise. Since food security policy is a national policy, the call for participation will be open for participation in all Canadian provinces and territories. This would allow for a representation of the many realities related to the many different contexts (geographical, political, economic, and cultural) related to food security nationwide. Canada also has two official languages, English and French that will be represented in the project. The research strategy will involve conducting two studies, one in each official language conducted using the exact same procedures.

For each study (English and French) we will seek to form two groups of participants: a lived experience (LE) group and a service provider (SP) group. Some considerations were involved in the creation of two distinct groups. First, there is a need to have a safer space between the participants in order for them to express their creativity and feel confident to voice their ideas. We believed that having same participant-type together promote this atmosphere of confidence required for the expression of creativity. Additionally, individuals with LE self-identifying as 2S/LGBTQI+ communities are looking for self-affirmative, inclusive, safe environments in which they are welcomed to express their issues and voice their ideas for change. We will be having, at first, distinct co-design workshops with two different groups of participants. We felt that having two groups of participants recognizes and supports these needs. Second, there are power dynamics at stake in this project that need to be recognized. Despite the fact that mixing people in groups of diverse experiences and expertise can be enriching for the codesign process, these disparities entail a risk that more dominant voices, sometimes those with more formal power or privilege, might overshadow others. Creating two distinct groups cannot ensure that all participants have an equal opportunity to contribute and be heard but can reduce unconscious biases and stereotypes that might arise in mixed groups, allowing for a more respectful and supportive environment for all participants. In that regards, the team is sensitive to the fact that people sometimes embody multiple identities, and that some individuals may self-identify as service providers and may also self-identify as members of the 2S/LGBTQI+ community and/or may also have experiences of food insecurity.

Accordingly, the participants will be invited to choose the group that best represents their experiences and expertise as well as choosing the group in which they feel more comfortable contributing. In the beginning of the co-design process with the design thinking approach, we will be seeking diversity of points of view and gathering different insights as much as possible. Having two groups will help to broaden the information collected and the understanding of the issue from two perspectives. Later in the codesign process, participants of both groups will be invited to take part simultaneously in a final workshop if they feel comfortable doing so. This will allow for merging and mixing the expertise of all participants and contribute to the enrichment of the data.

For the LE group, we aim to recruit individuals self-identifying as 2S/LGBTQI+ and individuals with a lived experience of food insecurity – or both (*n* = 4–7). For the SP group, we aim to recruit the same amount of participants (*n* = 4–7) working/volunteering in the food security network (*n* = 2–3); health professionals concerned with food security - dieticians, nutritionists, nurses, occupational therapists, social workers, general practitioners, etc. (*n* = 1–2); policy or government representatives and academics in the fields of dietetics and nutrition, public health, health promotion, and EDI (*n* = 1–2); Potential participants may have self-identified with one or more of these categories. All interested participants self-identifying with one or more of these categories will be eligible. However, a greater representation will be given to participants with lived experience. Eligibility criteria in terms of age and language will be the same for all: age of majority according to the province or territory regulations and being able to communicate in English or French. Although this project is not an Indigenous research project as it is defined by Canadian funding agencies (41), we do however aim to reach Indigenous individuals, communities and organizations. The team will deploy efforts to circulate the call for participation in their network of collaborators involved with Indigenous communities. A diverse representation of gender and sexualities, along with ages and ethnicities, will be sought to reach approximately 10 to 15 participants, for each study (English and French), for a total of 20 to 30 participants.

### 2.4 Study design and activities

The design and activities linked to design thinking mobilized in this protocol require flexibility (37). The activities will be iterative, alternating between divergent and convergent thinking, namely generating multiple ideas and then selecting the best ideas to come to a decision (36, 42). This process requires adjustments by the team to adapt to emerging creative insights, allowing for flexibility in ideation throughout the process (37). Therefore, the protocol outlined in this paper highlights the key processes of the co-design approach used; however, modifications may take place to maintain the flexibility required for the ideation process (39).

**Figure 1:**
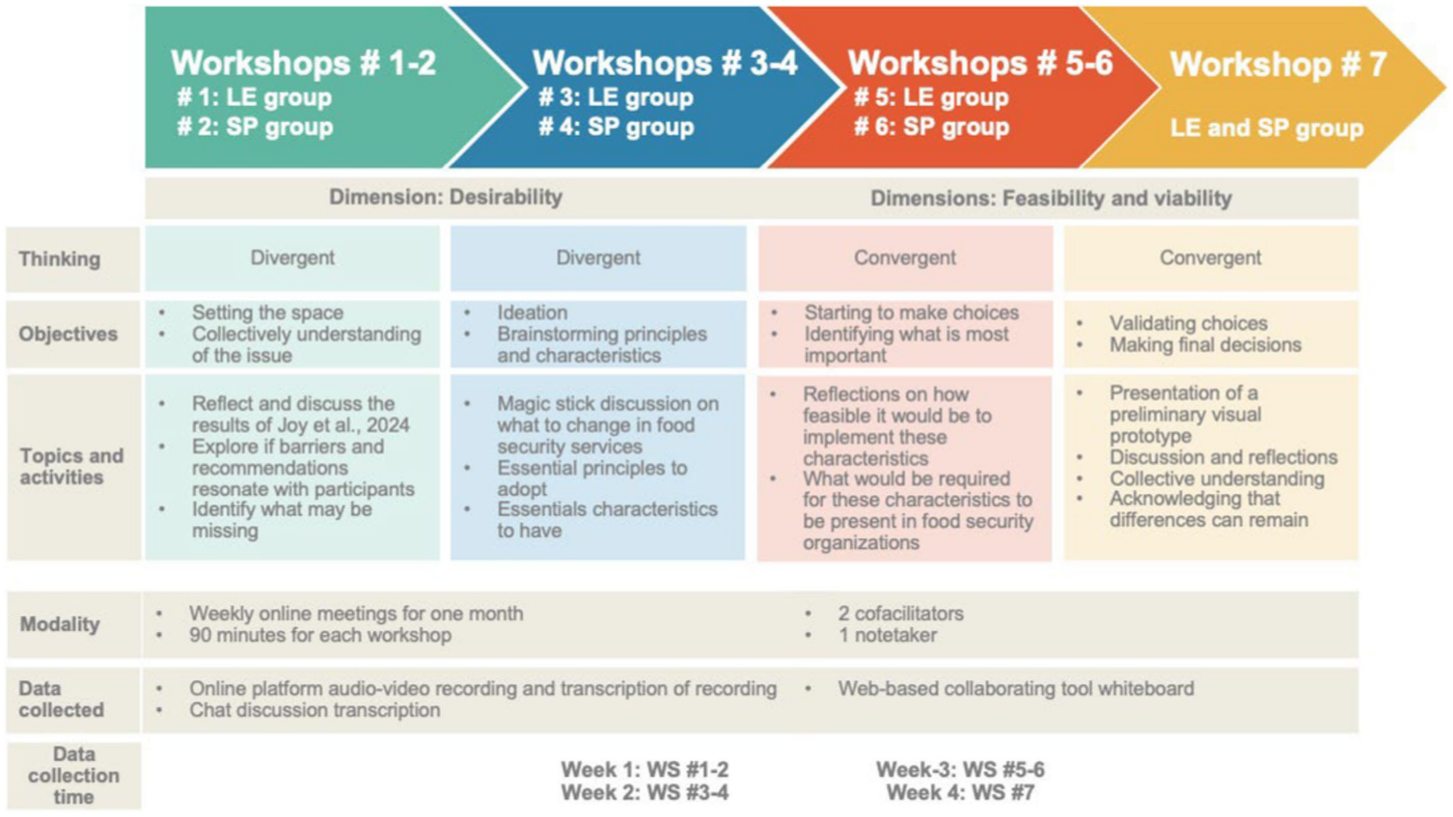
Representation of the workshops for each group of participants.

The activities will take place during a set of seven national online workshops of 90 min. A set of six workshops will take place as follows: three workshops with the LE group of participants and three workshops with the SP group of participants. A seventh and final workshop will convene both LE and SP participants. All workshops will be facilitated by the principal investigator (author #1) and a member of the partner organization (author # 4). The co-design workshops will follow a structure of divergent-convergent thinking. Divergent thinking will serve to generate multiple ideas for exploratory purposes regarding food security models, and convergent thinking will serve to choose and deepen the best ideas, then make choices about an ideal model of services. Figure 1 presents a visual representation of the workshops for all groups of participants and this will be conducted for both English and French study.

#### 2.4.1 Workshops #1–2: divergent thinking

For workshops #1–2, the participants will start by getting to know each other and setting up their safer space and terms of engagement collectively. Then, the facilitators will present the results of the previous community-based participatory research that explored food security access with 2S/LGBTQI+ communities (5). Among these results are the barriers to successfully accessing food support services, namely fear of discrimination, accessibility of the services, and quality of food offered. Other results pertain to recommendations to improve access to food support services, such as expanding opening hours, ensuring consistent training on 2S/LGBTQI+ communities and other minority groups, and engaging with 2S/LGBTQI+ communities to develop queer-friendly services. The participants will be invited to reflect and discuss these results for a deepener collective understanding. Then, the facilitators will invite the participants to share their ideas around recommendations that arise from the results. They will be invited to explore those recommendations and to reflect and share what resonates and what new ideas they might have. Discussions will involve visual, written, and verbal information exchange between the participants and the facilitators during and after the workshops, using the online collaboration tool whiteboard. This workshop structure will be conducted with the LE group (workshop #1) and with the SP group (workshop #2), independently. After both groups attended both workshops, the facilitators will look for similarities and distinctions.

#### 2.4.2 Workshops #3–4: divergent thinking

For workshops #3–4, the participants will be invited to deepen the initial discussions and reflections of workshops #1–2 by taking part in a “magic stick” discussion in which they will be invited to present what they would change if they had a magic stick to transform food security services in order for 2S/LGBTQI+ communities’ needs to be acknowledged and supported. The facilitators will invite participants to freely brainstorm around principles and characteristics they would like to experience or improve in the various food security services that exist. This activity will allow participants to create and imagine any features they would like, without censoring themselves. Various tools and strategies will be used to engage optimal participation of all participants: collective discussions (to support voicing ideas out loud), online collaboration tools like whiteboard (for visual support of ideas shared on virtual sticky-notes), online chat discussions (for written support for ideas shared). Again, this workshop structure will be conducted with both groups of participants, independently (workshop #3 with LE and workshop #4 with SP) and after both groups attended workshops #3–4, the facilitators will look for similarities and distinctions between the two groups. Based on the transcription and on the online visual collaborating tool used during the workshops, the facilitators will proceed to a first level of analysis to identify common elements of the characteristics and principles discussed by the participants. They will then work on a provisional textual and visual synthesis of the food security model and framework that embodies the characteristics and principles highlighted by the participants, which will be presented in the following workshops.

#### 2.4.3 Workshops #5–6 convergent-thinking

For workshops #5–6, the visual synthesis of the characteristics of a provisional model and framework principles for 2S/LGBTQI+ inclusive food services will be presented to the participants (based on workshops # 1 to 4). The synthesis will serve as a prompt to initiate the convergent-thinking process. The facilitators will guide the participants in offering free commentary, generating thoughts, and sharing ideas along with reflective questions regarding the choice of characteristics the model should have. Elements of feasibility and viability will be considered in this workshop. The facilitators will invite participants to reflect and share their ideas about how feasible it would be for different types of organizations involved in food security to embody and implement these characteristics. Guiding questions will revolve around what would be required for these characteristics to be present in food security services, what type of resources (financial, strategic, human) would be required for these characteristics to be implemented in organizations. Participants will be invited to comment on the characteristics they wish to keep and the ones they want to edit and the rationale using online collaborative tools. This will help to engage participants in a collaborative decision process. Achieving a collective understanding on the characteristics of this model and framework principles aligns with the importance of shared experience in this particular phase of the co-design process. Again, this workshop structure will be followed with both groups of participants, independently, and after both groups attended the workshop, the facilitators will look for similarities and disparities. The facilitators will then work on sketching a prototype of the service model and framework that would be presented to both groups of participants in workshop #4.

#### 2.4.4 Workshop #7 convergent-thinking

For workshop #7, participants of both groups (LE and SP) will be invited to take part to the workshop simultaneously, if they are comfortable. All participants wishing to be involved in workshop #7 will be welcomed to do so. They will be offered a preliminary visual presentation of the prototype of the food security service model and framework that embodies all the characteristics both groups would have chosen and agreed on, along with areas of disparities. All the participants will be invited to discuss and finalize key characteristics of the model. Particular attention will be provided by the facilitators to seek equal participation from both groups and ensure there will be less hierarchy possible in this co-design workshop. After workshop #7, the facilitators and research team will proceed to data analysis and will finalize the visual presentation of the service model and framework principles. The service model and framework principles will be presented back to a sub-group of participants of both groups (LE and SP) for a final validation using an online form questionnaire, written or verbal feedback, according to participants’ preferences.

### 2.5 Data collection

Multiple types of data will be collected in this project, including audio, textual, and visual content. These diverse formats support inclusivity, participation, and engagement among all participants. By offering various options for expression (speaking out loud with camera on or off, chatting and texting) participants can share their ideas in the most comfortable and coherent way for them. These accommodations also acknowledge potential invisible disabilities along with the varying levels of access to technology (Internet connection, computer access, smartphone) across the population, particularly among individuals from polymarginalized groups. Providing different ways to participate (by phone, computer, by texting, chatting etc.) can potentially overcome invisible disabilities and can help mitigate potential technology access issues faced by some participants.

The workshops will be audio-video recorded, and an automatic transcription will be generated with the online meeting platform. The research team will edit the automatically generated transcriptions for accuracy of content and anonymization of the data. The chat discussion of the online meeting platform will also be transcribed and anonymized to complete the audio transcription of the recording. The participants will also be offered the option to send text messages with their ideas to one of the facilitators and these will also be transcribed and anonymized to be included as data. The online collaborating tool used for each workshop (whiteboard) will also be included as textual and visual data to be analyzed by the team. All of these material will constitute the dataset. After each workshop, participants will be provided with a summary of the minutes of the workshop for validation purposes.

### 2.6 Data security and management

To ensure the security of the data, all the data will be stored on a secured online drive provided by the PI’s university. A copy of the data will also be stored on the PI’s external drive also protected by a password. Access to the files will be granted only to team members and protected by a password known only to them. All original files from the online meeting platform and web-based collaboration tool will be removed after each workshop. A data management plan will be created and circulated among the team.

### 2.7 Data analysis

The data will be subjected to a thematic analysis for design thinking (43). Thematic analysis is recognized as an approach that is deliberative (calling for discussion between various perspectives among the team) and reflective allowing for the integration of different theoretical perspectives (44). This analytical approach allows researchers to deeply explore peoples’ experiences and realities (45). The research team will go through the material multiple times (reading workshops and chat discussion transcriptions, reading the visual data like whiteboards, etc.) looking for patterns in the perspectives and experiences of the participants. The initial process of thematic analysis will involve individual review of the material by creating reflexive annotations, tags, and initial codes through the material by the researchers. Then, the research team will meet for a few rounds of collective reflection and discussion around the codes they initially gathered until complete coding of the data is achieved. After coding of the material is completed, the team will meet a few times to create the overarching themes arising from the data material. Meetings and discussions will take place until a consensus is reached among the team members.

## 3 Discussion

The objectives of this study protocol are to present the research activities and strategies of a co-design study aiming to enhance safety and inclusivity of food security services with and for 2S/LGBTQI+ individuals, and to concretely identify how to improve food security services with and for 2S/LGBTQI+ communities. In order to achieve these objectives, participatory co-creation activities and processes are used in a co-design approach mobilizing design thinking. Such approaches and processes focus on end users’ involvement all through the process. In the specific context of this project, we go beyond focusing on end-users. We focus on involving all potential individuals concerned about more inclusive food security, namely individuals from 2S/LGBTQI+ communities who are experiencing or have experienced food insecurity, service providers involved in the area of health, policy work, and food security. Involving all these individuals allows for the recognition and valuing of as many perspectives and experiences as possible to improve food security services with and for 2S/LGBTQI+ folks. The involvement of these partners in a project that aims to co-design an innovative prototype service model and framework represents, to our knowledge, a first-of-its-kind initiative.

### 3.1 Next, steps and expected results

This project constitutes a first step toward future extended reflections and action toward more inclusive, safe, and self-affirmative food security services. With the codesigned prototype service model and framework principles, the team envisions to circulate it at a provincial and national levels to food security organizations and other groups involved in 2S/LGBTQI+ health and inclusion. Circulating the service model and framework to both organizations involved in food security and individuals with lived experience should be sought. Gathering such diverse experiences of gender and sexualities nationwide may provide enriching improvements. This would allow for the model to be discussed, improved, and validated extensively for eventual broader dissemination. Potential methodologies that could be used for that matter may be based on qualitative data coupled to quantitative ones like Delphi survey. This can contribute to the creation of a sound logic model and theory of change highlighting expected outcomes of such innovative model of service. Using the similar participatory practices like the ones used in this protocol study, and specifically involving partners and end-users, this would favor the uptake and applicability of the model in formal food security services or lead to the creation of novel services that would reflect more inclusion and partnership with 2S/LGBTQI+ communities.

### 3.2 Potential challenges and reflexivity

This study protocol may present challenges and reflexivity. In terms of challenges, gathering participants with various experiences and expertise in a co-creation process implies careful reflection when designing the activities in order for all to contribute equally and in a meaningful, respectful, and inclusive manner. Another challenge is the needed methodological flexibility required in such a process. Finding this flexibility is key for ideation to happen and for creative insight to be fostered (38). However, adjusting the planned co-design process can be difficult to do in a pragmatic manner, namely understanding which aspects to adapt and how. For example, the study protocol is planned over 4 weeks. Spreading those 4 weeks apart will be key to offer sufficient time between the sessions for the facilitators to go through the data and present a synthesis to the participants. However, the exact time frame required to spread out the workshops can be hard to find and implies methodological flexibility. Also, the protocol unravels over seven workshop sessions, however, the process can reveal that the possibility to plan more sessions can be valuable. Additional workshop sessions can offer participants the opportunity to explore more ideas and can allow more time to make choices out of their ideation activities, if required.

In terms reflexivity, as this protocol gets finalized and reaches the point where the studies will be ready to be conducted, learnings will happen along the way. These learnings can lead to insightful reflections around tools and practices to facilitate optimal participation and processes and decision-making to integrate flexibility in the methodological process. The involvement of a design expert in the elaboration of this protocol supported these reflections. We will plan for sustained involvement of a design expert when conducting the two studies in English and French. Sustained involvement of design experts can deepen the reflections on dividing groups of participants according to their experience and expertise and identify safe and inclusive ways of bringing the groups together during the last stages of the process along with rigorous adaptation of the process. Some questions can guide the learning process, like how to facilitate discussion and creativity, what does an online safe space look like for the participants, and what was the participants’ experience of the project, for example.

## 4 Conclusion

In this research protocol paper, we present the co-design approach mobilizing design thinking that will be used to rethink food security with and for 2S/LGBTQI+ communities. Subsequently, we will report on the results of this co-design research, potentially highlighting the principles and characteristics that would make food security services safer and more inclusive for 2S/LGBTQI+ communities. We look forward to using these findings to adapt, validate, and disseminate an innovative food security service model and framework principles that organizations can use to improve or transform their practices.
